# Age-Specific Risk of High-Grade Cervical Lesions in Unvaccinated Women: Implications for HPV Screening Policies in Low-Vaccination Settings

**DOI:** 10.3390/diagnostics16050659

**Published:** 2026-02-25

**Authors:** Süleyman Özen, Eda Güner Özen, Muzaffer Sancı

**Affiliations:** 1 Department of Gynecologic Oncology, İzmir City Hospital, İzmir 35540, Turkey; drsanci@yahoo.com; 2 Department of Obstetrics and Gynecology, İzmir City Hospital, İzmir 35540, Turkey; edaguner92@gmail.com

**Keywords:** cervical intraepithelial neoplasia (CIN2+), cervical screening, cytology, high-risk HPV, HPV persistence, HPV vaccination, unvaccinated women

## Abstract

**Background:** In Turkey, where HPV vaccination is not yet routinely implemented, cervical cancer screening with primary HPV testing begins at age 30. This may result in undetected high-grade cervical lesions in younger unvaccinated women. The aim of this study was to evaluate the age-specific prevalence of CIN2+ lesions, HPV persistence, and the diagnostic performance of cytology among women under 30. **Methods:** A retrospective cohort study was conducted in 689 unvaccinated women aged 18–30 who underwent colposcopy following a positive high-risk HPV (hrHPV) test. Participants were stratified into two groups: 18–24 and 25–30 years. HPV genotypes, 12-month persistence, cytological findings, and biopsy-confirmed histopathology were analyzed using logistic regression and ROC curve analysis. **Results:** CIN2+ lesions were identified in 17.5% of women aged 18–24 and 44.9% of those aged 25–30 (*p* < 0.001). Persistent HPV 16 infection was the strongest predictor of CIN2+. Cytology showed low sensitivity in detecting CIN2+ (36.4% and 47.2% in the respective age groups). ROC analysis revealed better model performance in older women (AUC 0.81 vs. 0.73, *p* = 0.047). **Conclusions:** A significant proportion of unvaccinated women aged 25–30 harbor undiagnosed CIN2+ lesions. These findings support lowering the age of HPV-based screening to 25 in low-vaccination settings. Cytology alone is insufficient as a triage tool in this population.

## 1. Introduction

Cervical cancer remains one of the most preventable yet persistently burdensome malignancies affecting women worldwide. According to global cancer statistics, more than 600,000 new cases and over 340,000 deaths were reported in 2020, with the majority occurring in low- and middle-income countries [1]. The widespread implementation of HPV vaccination and organized screening programs has led to substantial reductions in cervical cancer incidence in many high-income countries; however, this success has not been uniformly achieved worldwide [2].

Persistent infection with high-risk human papillomavirus (hrHPV) is well established as the central etiologic factor in the development of cervical intraepithelial neoplasia (CIN) and invasive cervical cancer, with HPV genotypes 16 and 18 accounting for the majority of high-grade lesions and cancers [3]. On this basis, HPV-based screening strategies have increasingly replaced cytology-based approaches due to their superior sensitivity for detecting CIN2+ lesions [4].

Despite these advances, the optimal age to initiate HPV-based screening remains controversial. International guidelines generally recommend starting HPV testing at age 25 or later, citing the high prevalence of transient HPV infections and the low incidence of invasive cervical cancer in younger women, as well as concerns regarding overdiagnosis and overtreatment [5]. Nevertheless, accumulating evidence suggests that a subset of young women—particularly those with persistent hrHPV infection—may already harbor high-grade cervical lesions, challenging the adequacy of strict age-based screening thresholds [6].

In Turkey, the national cervical cancer screening program currently initiates primary HPV-based screening at age 30, and HPV vaccination has not yet been incorporated into the national immunization schedule [7]. As a result, a considerable proportion of young women remain both unvaccinated and outside organized screening programs. Notably, the cohort included in this study consisted of women under 30 years of age who underwent HPV testing outside the national program, most often in private laboratories at their own expense. These tests were commonly prompted by anxiety, the presence of genital warts in sexual partners, or a desire for reassurance prior to planned HPV vaccination, a pattern consistent with opportunistic HPV testing reported in other settings [8]. Women with positive HPV results were subsequently referred to our gynecologic oncology clinic for further evaluation.

The aim of this study was to compare HPV persistence, cytologic findings, and histopathologic outcomes in hrHPV-positive women aged 18–24 and 25–30 years who underwent colposcopic evaluation. By focusing on age-specific risk profiles in an unvaccinated population, this study seeks to provide evidence to inform optimization of cervical cancer screening strategies, particularly regarding the potential initiation of HPV-based screening at age 25 in Turkey.

## 2. Materials and Methods

This retrospective cohort study was conducted at İzmir City Hospital Gynecologic Oncology Surgery Clinic, a tertiary referral center, following approval from the institutional ethics committee (Approval No: 2025/228, dated 21 May 2025). The cohort consisted of women who presented to the gynecologic oncology surgery unit between December 2023 and September 2025 and were referred for colposcopic evaluation due to a positive HPV result. Women aged 18 to 24 years were included only if their HPV samples had been obtained either at outside centers prior to referral or by the obstetrics and gynecology department of the same institution. The study was designed to evaluate and compare colposcopic, cytologic, and histopathologic findings as well as 12-month HPV persistence rates in two age-based groups: women aged 18–24 years and those aged 25–30 years.

Inclusion criteria for both groups were: a positive high-risk HPV test result; availability of liquid-based cervical cytology (Pap test), HPV genotyping results, and colposcopic biopsy data, no previous diagnosis of HPV infection, abnormal cytology, or cervical intraepithelial neoplasia (CIN); no prior HPV vaccination, and no active smoking or family history of cervical cancer. Patients with immunodeficiency, pregnancy, incomplete follow-up data, or missing histopathology or HPV results were excluded from the study. All patients included in the analysis were immunocompetent and had no history of prior cervical interventions.

Patients were divided into two groups based on age at the time of initial HPV testing: Group 1 consisted of women aged 18–24 years, and Group 2 included women aged 25–30 years. Following initial triage and colposcopy, all patients were followed clinically and virologically. A repeat cotest (HPV test and cytology) was performed 12 months after the initial positive HPV result. HPV clearance was defined as a negative high-risk HPV result at 12 months, while persistence was defined as detection of the same high-risk HPV genotype after one year. Only patients with complete 12-month follow-up data were included in the persistence analysis.

Cytological samples were obtained using the ThinPrep™ liquid-based cytology system (Hologic, Inc., Bedford, MA, USA) and evaluated by experienced cytopathologists according to the 2014 Bethesda System. A single-reading approach was used, consistent with routine clinical practice; however, all interpretations were performed by board-certified cytopathologists with a minimum of five years of experience in cervical cytology interpretation. Results were classified as Negative for Intraepithelial Lesion or Malignancy (NILM), ASCUS, ASC-H, LSIL, or HSIL.

HPV genotyping was performed using the Roche Cobas 4800 system (Roche Molecular Systems, Pleasanton, CA, USA), which individually detects HPV 16 and HPV 18, and concurrently identifies a pooled group of 12 other high-risk HPV types (HPV 31, 33, 35, 39, 45, 51, 52, 56, 58, 59, 66, and 68), categorized as non-16/18 hrHPV. This method provided type-specific data necessary for analyzing HPV persistence and its association with cytologic and histologic outcomes.

Colposcopic examinations were performed by gynecologic oncologists with formal training in lower genital tract pathology and colposcopy. A standardized protocol was followed for all procedures, beginning with the application of 5% acetic acid to the cervix to identify acetowhite epithelium, mosaicism, punctation, atypical vascular patterns, and irregular lesion margins. Colposcopic findings were classified as normal or abnormal based on these criteria. Biopsies were obtained from any abnormal areas, and endocervical curettage (ECC) was performed when the squamocolumnar junction was not fully visible or endocervical involvement was suspected. All biopsy samples were evaluated by expert gynecologic pathologists at the same institution.

All statistical analyses were conducted using IBM SPSS version 28 Statistics for Windows (IBM Corp., Armonk, NY, USA). A *p*-value of <0.05 was considered statistically significant.

Descriptive statistics were used to summarize demographic and clinical data. Continuous variables such as age were reported as mean ± standard deviation (SD), while categorical variables (e.g., HPV genotype, cytology classification, histopathology results) were presented as frequencies and percentages.

To evaluate differences between the 18–24 and 25–30 age groups, Chi-square (χ^2^) tests were used for categorical variables. Kaplan–Meier survival analysis was performed to compare 12-month HPV clearance between groups, and the log-rank test was used to assess statistical significance in survival curves.

To quantify the magnitude of risk, relative risk (RR) and odds ratios (OR) with 95% confidence intervals (CIs) were calculated to assess the likelihood of CIN2+ lesions in the 25–30 age group compared to the 18–24 age group.

A multivariate logistic regression analysis was performed to identify independent predictors of CIN2+ histopathology. Covariates included age group (≥25 vs. <25), HPV genotype (e.g., HPV 16 vs. others), cytology result (normal vs. abnormal), and 12-month HPV persistence (positive vs. negative). Results were presented as adjusted odds ratios (aORs) with 95% CIs. Prior to model inclusion, multicollinearity was assessed using variance inflation factor (VIF) thresholds. Only independent and clinically relevant predictors were retained.

In addition, a stratified subgroup analysis was conducted to assess CIN2+ prevalence among HPV 16-positive patients in both age groups. This aimed to evaluate the specific contribution of high-risk genotypes to disease severity.

To improve model accuracy and address class imbalance, particularly in the 18–24 age group where CIN2+ was less frequent, the Synthetic Minority Over-sampling Technique (SMOTE) was applied before training a logistic regression model for CIN2+ prediction. The performance of this model was evaluated using a receiver operating characteristic (ROC) curve, and the area under the curve (AUC) was calculated to assess discriminative ability. To compare the diagnostic performance of the logistic regression models developed for each age group, ROC analysis was conducted, and the AUC was calculated for both models. To assess whether the difference in AUC values between the two groups was statistically significant, the DeLong test was employed. This nonparametric method allows for comparison of correlated ROC curves derived from models predicting the same binary outcome (CIN2+ vs. non-CIN2+).

Lastly, the diagnostic accuracy of cytology in detecting CIN2+ lesions was evaluated using a confusion matrix, from which sensitivity, specificity, positive predictive value (PPV), negative predictive value (NPV), and overall accuracy were derived. This enabled a direct assessment of the strengths and limitations of cytology as a standalone triage tool in both age groups.

The complete anonymized dataset supporting the findings of this study is available as Supplementary Materials, and a detailed description of all variables and coding definitions is provided in Supplementary Materials Table S1 and Table S2. 

All analyses were reviewed in collaboration with a clinical biostatistician to ensure methodological rigor and interpretative validity.

## 3. Results

A total of 689 women were included in the final analysis: 325 patients aged 18–24 years (Group 1) and 364 patients aged 25–30 years (Group 2). The mean age was 22.6 ± 1.49 years in Group 1 and 26.5 ± 1.16 years in Group 2. Demographic and clinical characteristics of both groups are presented in Table 1.

HPV 16 was the most commonly detected genotype in both groups, with a prevalence of 49.8% in Group 1 and 52.2% in Group 2 (*p* = 0.482). The distribution of HPV 18 and other high-risk genotypes did not differ significantly between groups. Multiple high-risk infections were present in 16.6% of patients in Group 1 and 17.0% in Group 2 (*p* = 0.904).

**Figure 1:**
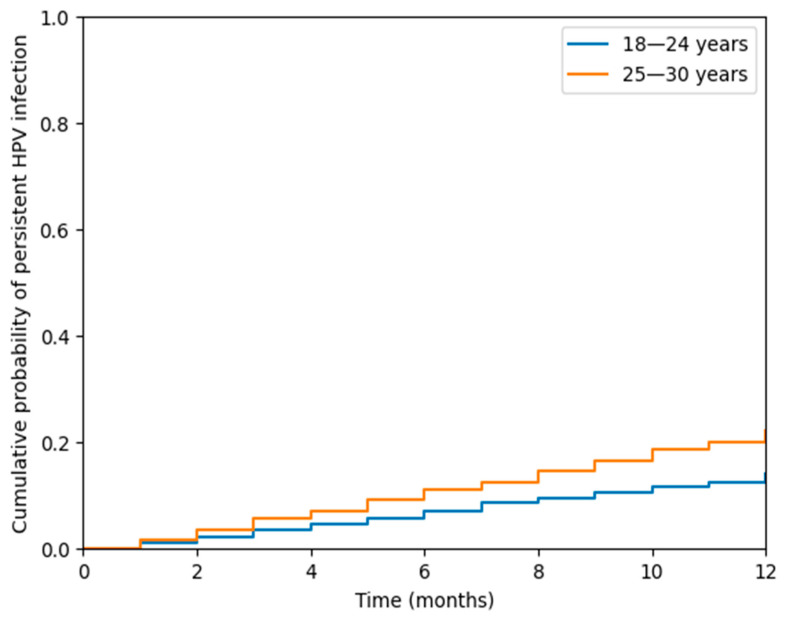
HPV Persistence over 12 Months.

Figure 1 illustrates 12-month HPV persistence rates in both age groups. At one-year follow-up, persistent high-risk HPV infection was significantly more frequent in women aged 25–30 years (Group 2) compared to those aged 18–24 years (Group 1) (22.0% vs. 14.8%, *p* = 0.0198). Kaplan–Meier time-to-event analysis, using HPV persistence as the endpoint, demonstrated a significantly higher cumulative probability of persistent infection in Group 2 (log-rank *p* = 0.021). These findings indicate that women aged ≥25 years exhibit a substantially increased risk of sustained HPV infection over 12 months.

Cytological analysis revealed that normal cytology (NILM) was more frequent in Group 2 than Group 1 (65.6% vs. 57.8%, *p* = 0.048). While ASCUS and LSIL findings were more prevalent in Group 1, high-grade abnormalities (HSIL/ASC-H) were significantly more common in Group 2 (3.3% vs. 0.3%, *p* < 0.001). A statistically significant association between HPV genotype and cytological abnormality was found in Group 2 (*p* < 0.001), but not in Group 1 (*p* = 0.371), indicating limited cytological predictive value in the younger cohort.

Colposcopic biopsy was performed in 122 women in Group 1 and 248 in Group 2. Histopathologic analysis revealed biopsy-proven CIN2+ lesions in 22 patients in Group 1 (18.0%) and 112 patients in Group 2 (45.1%), a difference that was statistically significant (*p* < 0.001). The relative risk (RR) of CIN2+ in Group 2 compared to Group 1 was 2.51 (95% CI: 1.65–3.83), and the odds ratio (OR) was 3.75 (95% CI: 2.11–6.66). Notably, CIN2+ lesions were identified in 7 patients in Group 1 and 56 in Group 2 despite normal cytology. Among these cytologically normal but histologically high-grade cases, HPV persistence at 12 months was observed in 42.9% of Group 1 and 60.7% of Group 2.

Table 2 shows the cytology performance for CIN2+ detection. Analysis restricted to patients who underwent colposcopic biopsy. In Group 1, sensitivity was 36.4%, specificity 78.0%, positive predictive value (PPV) 33.3%, negative predictive value (NPV) 80.2%, and overall accuracy 69.7%. In Group 2, cytology showed higher sensitivity (47.2%) but lower specificity (53.4%), with a PPV of 64.9%, NPV of 35.4%, and overall accuracy of 49.4%. ROC curve analysis of cytology alone in Group 1 yielded an AUC of 0.61.

Table 3 shows the logistic regression analysis for predictors of CIN2+. To better predict CIN2+ lesions, multivariate logistic regression models were developed for biopsy-confirmed cases of each group. In both models, the following variables were included: age group, HPV 16 positivity, high-grade cytology, and 12-month HPV persistence. All variables were independently associated with an increased risk of CIN2+. Specifically, in the pooled analysis, age ≥25 years (adjusted odds ratio [aOR] 2.55, 95% CI: 1.65–3.94, *p* < 0.001), HPV 16 positivity (aOR 2.91, 95% CI: 1.97–4.31, *p* < 0.001), HPV persistence (aOR 2.43, 95% CI: 1.61–3.68, *p* < 0.001), and high-grade cytology (aOR 2.07, 95% CI: 1.26–3.41, *p* = 0.004) were all significant predictors of CIN2+.

**Figure 2:**
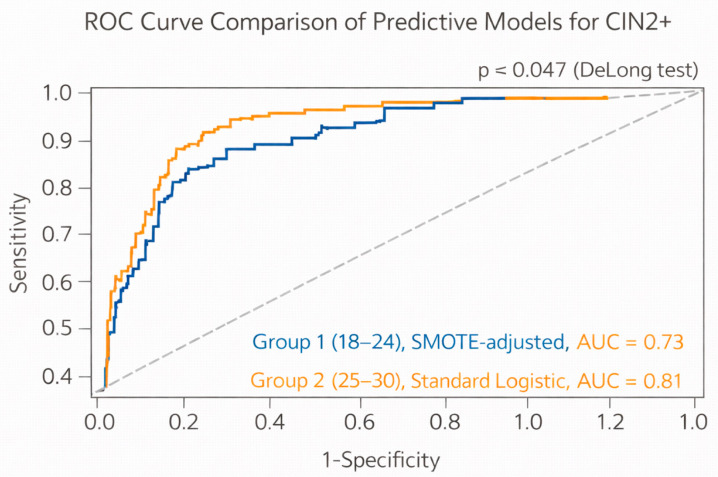
ROC Curve Comparison of Predictive Models for CIN2+.

Figure 2 shows the ROC curve comparison of predictive models for CIN2+. In ROC curve analysis of the multivariable models, the Group 1 model (developed using SMOTE to address class imbalance) demonstrated an AUC of 0.73, indicating acceptable discrimination. The Group 2 model, developed without oversampling, yielded a higher AUC of 0.81, reflecting good discriminatory performance. The DeLong test confirmed that the difference between the two AUC values was statistically significant (*p* = 0.047), suggesting that the prediction model in older women was significantly more effective in identifying CIN2+ lesions.

## 4. Discussion

In this study, we investigated the predictive utility of HPV triage and cytology among women aged 18–30, a population group where current screening guidelines offer differing recommendations. Our results highlight a significant burden of histopathologically confirmed CIN2+ lesions in both the 18–24 and 25–30 age groups (17.5% vs. 44.9%), despite cytologic normalcy in a substantial subset. These findings carry important implications for current screening thresholds, especially in settings such as Turkey, where HPV-based screening is initiated at age 30 [7].

Recent studies have reported varying CIN2+ prevalence rates in women aged 25–30, largely influenced by HPV vaccination status and screening protocols. For instance, the ATHENA trial documented a CIN2+ prevalence of approximately 19.4% among HPV-positive women aged 21–29, with lower rates observed in vaccinated subgroups [9]. Similarly, population-based data from Australia and China have reported CIN2+ rates ranging from 20% to 26% in women aged 25–30 undergoing HPV-based screening [8,10]. In contrast, our study found a notably higher prevalence of 44.9% in the same age group, which may be attributed to the absence of HPV vaccination, opportunistic screening patterns, and a higher proportion of persistent HPV 16 infections in our cohort. These differences underscore the elevated risk burden in unvaccinated populations and reinforce the importance of early detection strategies.

Population-level data from England have shown that initiating cervical screening at age 24.5, rather than 25, leads to a significant rise in the detection of FIGO stage I cervical cancers, with no corresponding increase in stage II or higher disease, suggesting enhanced early detection rather than overdiagnosis [11]. Our study offers clinical insight supporting these epidemiological trends: we observed a 44.9% prevalence of CIN2+ lesions in unvaccinated women aged 25–30, a considerable proportion of which were missed by cytology. These findings echo the implications of Castanon and Sasieni’s analysis—that deferring screening to age 30, as currently practiced in Turkey, may delay diagnosis in women who are already at risk. In settings lacking universal HPV vaccination, early screening may serve as a crucial opportunity for intervention. Our results support a policy shift to initiate HPV-based screening at age 25, particularly in countries where high-grade precancerous lesions remain prevalent and cytology alone offers limited diagnostic yield.

The detection of CIN2+ lesions in 17.5% of women aged 18–24 and 44.9% of those aged 25–30 is consistent with earlier studies indicating that HPV infection and early neoplastic transformation can occur in women younger than traditional screening cut-offs [3]. In our cohort, persistent HPV 16 infection at 12 months was strongly associated with CIN2+ development, confirming results from Schiffman, who emphasized the high carcinogenic potential of HPV 16 even in younger women [12].

Given the limited diagnostic performance of cytology alone in detecting CIN2+ as evidenced by its sensitivity of 36.4% in Group 1 and 48.3% in Group 2—and the high rate of occult high-grade lesions in women with normal cytology, initiating HPV screening at age 25 could allow for more timely detection and intervention. Similar to our findings, the ATHENA trial reported that cytology alone missed approximately 40% of CIN2+ lesions in women aged 21–29, with HPV testing significantly improving detection sensitivity [9,10]. In our cohort, cytology failed to detect high-grade lesions in 9.6% of Group 1 and 15.7% of Group 2 patients, echoing concerns from Fontham et al. regarding the limitations of cytology-first approaches [5,13].

ROC analysis of our predictive models showed acceptable discriminatory power, with AUCs of 0.73 and 0.77 for Groups 1 and 2, respectively. These are comparable to values reported by Wentzensen et al. in models integrating cytology, HPV genotype, and persistence [14]. The DeLong test demonstrated a statistically significant difference between age groups, suggesting that predictive models may perform better in slightly older women where lesion biology is more advanced and persistence patterns are clearer.

According to the latest national cancer statistics in Turkey, cervical cancer does not rank among the top ten malignancies in women aged 15–24; however, it constitutes approximately 5.1% of all female cancers in the 25–49 age group [7]. This epidemiologic trend mirrors our findings, in which CIN2+ lesions were relatively uncommon in women aged 18–24 but substantially more prevalent in those aged 25–30, with a statistically significant difference between the groups (17.5% vs. 44.9%, *p* < 0.001). Furthermore, our multivariate analysis identified persistent HPV infection—particularly with genotype 16—as the most significant predictor of high-grade lesions in both cohorts. These findings suggest that while routine screening may not be warranted under age 21, the rising incidence of cervical pathology beginning at age 25 warrants earlier HPV-based screening.

Our findings also carry direct relevance to national policy. In Turkey, cervical cancer incidence begins to rise after age 25 and peaks between 35 and 44 years [7]. Thus, waiting until age 30 to begin HPV-based screening may delay detection in women already at risk. Shifting the screening initiation to age 25 could reduce this diagnostic gap, especially in unvaccinated populations [15].

It is also important to contextualize our data within the broader landscape of HPV vaccination. Unlike many high-income countries, Turkey does not yet include HPV vaccination in its national immunization schedule. Consequently, our cohort—comprising entirely unvaccinated women—may reflect a more accurate risk profile for screening policy in non-immunized settings. Integrating the vaccination drastically reduces HPV 16/18 prevalence and subsequent high-grade lesions, allowing for safer delays in screening in vaccinated cohorts [16,17,18].

Finally, our study provides real-world evidence that HPV persistence is a critical metric for risk stratification across all age groups. While we acknowledge that overdiagnosis and overtreatment remain concerns—particularly in women under 25—our findings support the selective use of HPV triage and closer surveillance in those with persistent high-risk genotypes, especially HPV 16. This approach aligns with risk-based management principles now recommended by updated ASCCP guidelines and adopted in multiple global programs [19].

This study has several notable strengths. First, it addresses a critical gap in the literature by evaluating the diagnostic performance of HPV triage and cytology in women aged 18–30, including a subgroup (18–24 years) not routinely covered by current screening guidelines [19]. The inclusion of histologically confirmed CIN2+ as the primary endpoint enhances clinical relevance. Moreover, the study population was carefully selected to reduce confounding, excluding individuals with prior abnormal cytology, smoking, immunosuppression, or HPV vaccination. The use of robust statistical methods including logistic regression modeling, ROC curve analysis, and DeLong testing—adds methodological rigor, while the application of SMOTE addressed class imbalance effectively in predictive modeling.

However, certain limitations must be acknowledged. The retrospective design introduces inherent risks of selection and information bias, particularly in the younger cohort where HPV testing was not universally applied. Cytology and histopathology assessments were not centralized; specimens were interpreted by different pathologists across institutions, which may have led to interobserver variability. Additionally, the absence of HPV-vaccinated individuals limits the generalizability of findings to populations with high immunization coverage. Finally, as this study was based on a single-center cohort, multicenter prospective validation is needed before policy-level recommendations can be generalized.

## 5. Conclusions

Our findings demonstrate that a considerable proportion of women under the age of 30 particularly those aged 25 to 30 harbor high-grade cervical lesions, many of which are not detected by cytology alone. The limited sensitivity of cytology and the strong association between HPV persistence, especially with genotype 16, and CIN2+ underscore the need for more effective early detection strategies. In unvaccinated populations such as Turkey, where HPV-based screening currently begins at age 30, these results support lowering the screening initiation age to 25.

Given that cervical cancer incidence in Turkey begins to rise after age 25, earlier implementation of HPV-based primary screening would allow timely identification and management of at-risk individuals, ultimately reducing the progression to invasive disease. Furthermore, our findings highlight the importance of including HPV vaccination in the national immunization program. Broader vaccination coverage, combined with age-appropriate screening strategies, is essential for sustainable cervical cancer prevention in the coming decades. National policies should urgently be updated to reflect these dual priorities.

## 6. Patents

The authors declare that there are no patents resulting from the work reported in this manuscript.

## Figures and Tables

**Table 1:** Demographic and Clinical Characteristics of the Study Population.

Parameter	Group 1 (18–24 Years)	Group 2 (25–30 Years)	*p*-Value
n	325	364	—
Mean age (years, ±SD)	22.6 ± 1.49	26.5 ± 1.16	<0.001
HPV 16 (%)	49.8%	52.2%	0.482
HPV 18 (%)	9.8%	11.3%	0.562
Other hrHPV (%)	34.5%	38.5%	0.288
Multiple infections (%)	16.6%	17.0%	0.904
HPV persistence at 12 mo (%)	14.8%	22.0%	0.0198 *
Cytology—NILM (%)	57.8%	65.6%	0.048 *
Cytology—ASCUS (%)	23.1%	22.0%	0.724
Cytology—LSIL (%)	16.6%	6.9%	<0.001 *
Cytology—HSIL/ASC-H (%)	0.3%	3.3%	<0.001 *

* *p* < 0.05.

**Table 2:** Cytology Performance for CIN2+ Detection.

Metric	Group 1	Group 2
Sensitivity (%)	36.4	47.2
Specificity (%)	78.0	53.4
Positive Predictive Value (%)	33.3	64.9
Negative Predictive Value (%)	80.2	35.4
Diagnostic Accuracy (%)	69.7	49.4
AUC (Cytology only)	0.61	0.66

**Table 3:** Logistic Regression Analysis for Predictors of CIN2+ (Multivariate Model).

Variable	Adjusted Odds Ratio (aOR)	95% Confidence Interval	*p*-Value
Age ≥ 25 years	2.55	1.65–3.94	<0.001
HPV 16 positivity	2.91	1.97–4.31	<0.001
HPV persistence at 12 months	2.43	1.61–3.68	<0.001
High-grade cytology (HSIL/ASC-H)	2.07	1.26–3.41	0.004

## Data Availability

The datasets generated and/or analyzed by this research are included in this paper and are publicly available in the Zenodo repository https://zenodo.org/records/18139434 (accessed on 3 January 2026).

## Supplementary Materials

The following supporting information can be downloaded at: https://www.mdpi.com/article/10.3390/diagnostics16050659/s1, Table S1: Group 1 under 25 years old; Table S2: Group 2 over 25 years old.

## Abbreviations

The following abbreviations are used in this manuscript: HPV: Human papillomavirus; CIN2+: Cervical intraepithelial neoplasia grade 2 or higher; hrHPV: High-risk human papillomavirus; NILM: Negative for Intraepithelial Lesion or Malignancy; ASCUS: Atypical squamous cells of undetermined significance; ASC-H: Atypical squamous cells, cannot exclude HSIL; LSIL: Low-grade squamous intraepithelial lesion; HSIL: High-grade squamous intraepithelial lesion; ECC: Endocervical curettage; ROC: Receiver operating characteristic; AUC: Area under the curve; SMOTE: Synthetic Minority Over-sampling Technique; aOR: Adjusted odds ratio; CI: Confidence interval.
